# ERFVII transcription factors and their role in the adaptation to hypoxia in Arabidopsis and crops

**DOI:** 10.3389/fgene.2023.1213839

**Published:** 2023-08-15

**Authors:** Elena Loreti, Pierdomenico Perata

**Affiliations:** ^1^ Institute of Agricultural Biology and Biotechnology, CNR, National Research Council, Pisa, Italy; ^2^ PlantLab, Center of Plant Sciences, Sant’Anna School of Advanced Studies, Pisa, Italy

**Keywords:** crops, ethylene response factor, flooding, hypoxia, rice, submergence, transcription factor

## Abstract

In this review, we focus on ethylene transcription factors (ERFs), which are a crucial family of transcription factors that regulate plant development and stress responses. ERFVII transcription factors have been identified and studied in several crop species, including rice, wheat, maize, barley, and soybean. These transcription factors are known to be involved in regulating the plant’s response to low oxygen stress—hypoxia and could thus improve crop yields under suboptimal growing conditions. In rice (*Oryza sativa*) several *ERFVII* genes have been identified and characterized, including *SUBMERGENCE 1A* (*SUB1A*), which enables rice to tolerate submergence. The *SUB1A* gene was used in the development of SUB1 rice varieties, which are now widely grown in flood-prone areas and have been shown to improve yields and farmer livelihoods. The oxygen sensor in plants was discovered using the model plant Arabidopsis. The mechanism is based on the destabilization of ERFVII protein via the N-degron pathway under aerobic conditions. During hypoxia, the stabilized ERFVIIs translocate to the nucleus where they activate the transcription of hypoxia-responsive genes (*HRG*s). In summary, the identification and characterization of ERFVII transcription factors and their mechanism of action could lead to the development of new crop varieties with improved tolerance to low oxygen stress, which could have important implications for global food security.

## Introduction

Transcription factors (TFs) are proteins that can specifically bind to cis-acting elements in the promoter region of eukaryotic genes in order to regulate gene expression (Lelli et al., 2012; Lambert et al., 2018). They are protein-coding genes and account for around 4%–10% of the genomes of all species (Babu et al., 2004). More than 9% of total protein-coding genes in *Arabidopsis thaliana* belong to TFs (Swarbreck et al., 2008; Pruneda-Paz et al., 2014). TFs are also central mediators of cellular response to different biotic and abiotic stress due to their involvement in the transcriptional control of stress-responsive genes.

Research is increasingly focused on identifying new transcription factors aimed at revealing plant responses to stressful conditions. Many classes of TFs have been identified, such as bZIP, MYB, MADS, NAC, and WRKY (Qu and Zhu, 2006). In this review we highlight the role of ERFVII proteins in low oxygen conditions, a stress that plants are often faced with. The ERF family encode transcription factors with several functions, ranging from plant development and physiology (Nakano et al., 2006). A computational analysis identified 122 and 139 ERF family genes in Arabidopsis (*Arabidopsis thaliana*) and rice (*Oryza sativa* L. subsp. japonica), respectively (Nakano et al., 2006). ERF proteins were originally isolated as transcription factors regulating stress-responsive genes, including pathogen infection, salt stress, osmotic stress, wounding, drought, hypoxia, temperature stresses (Licausi et al., 2013). Nakano et al. (2006) proposed a classification of the ERF family which recapitulates the phylogenetic history of this class of transcription factors and also allows proteins possessing similar regulatory features to be grouped (Nakano et al., 2006; Licausi et al., 2013).

Plants are sessile organisms that are not able to escape from adverse environmental conditions. Although oxygen availability is essential for their survival, plants are frequently faced with low oxygen stress due to variations in the environment where they live, such as sudden floods, severe rainfall as well as low oxygen originating at high altitudes (Abbas et al., 2022). These adverse conditions lead to hypoxia, which negatively affects plant growth and crop production (Bailey-Serres and Voesenek, 2010; Loreti et al., 2016; Loreti and Perata, 2020).

In higher plants, oxygen deprivation compromises most cellular functions, including mitochondrial respiration, ATP production, and energy supply, which can lead to death (Zabalza et al., 2009). Without oxygen, the reduced compounds produced in the glycolysis and in the Krebs cycle cannot be re-oxidized through the electron transport chain. This results in the switch to the fermentative pathway with the re-oxidation of the NADH produced by the glycolytic pathway and some ATP production. When hypoxia occurs, plants respond by reprogramming their metabolism though the expression of a specific set of genes that help overcome unfavorable environmental conditions (Loreti et al., 2005; Lasanthi-Kudahettige et al., 2007; Mustroph et al., 2010; Narsai et al., 2011).

Nearly all crops are negatively impacted by waterlogging and flooding in terms of growth and seed production, leading to large losses of agricultural productivity and thus hampering food security. In the last few years in the United States, drought has been the main cause of losses in crop production, followed by flooding (Bailey-Serres et al., 2012). In Europe, increased rainfall intensity has caused numerous flooding events with dramatic economic consequences (Olesen et al., 2011). The negative effect of flooding on plant performance is caused by the associated reduction in tissue oxygen. Rice is the only crop species that can survive prolonged periods of submergence.

## Rice tolerance relies on SUB1A, an ERFVII transcription factor

**FIGURE 1:**
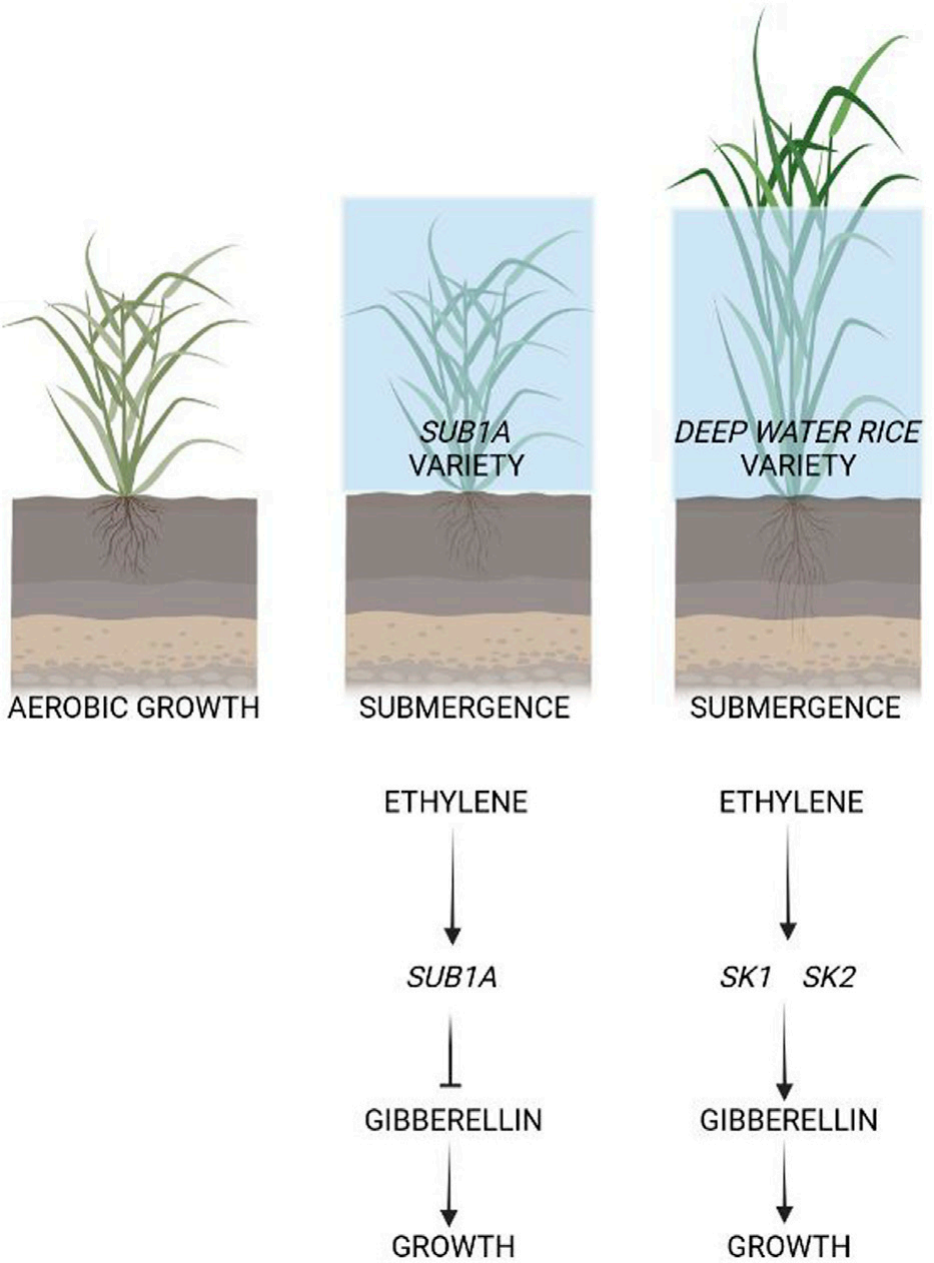
Adaptation of rice varieties to submergence. Rice varieties possessing the *SUB1A* gene are able to survive prolonged submergence because ethylene, which is entrapped between the plant-water interface, activates the transcription of the *SUB1A.* Through a series of different steps, *SUB1A* then inhibits the growth of the plant so that the energy consumption and depletion of carbon reserves is limited to basic cell housekeeping and is not wasted in an attempt to grow more rapidly than the rise in water level. This enables these varieties to survive for relatively long periods of submergence. The deep-water varieties, instead, possess the *SNORKEL* genes (*SK1* and *SK2*) which, acting in an opposite direction to SUB1A, promote a very fast stem elongation. These genes are similarly induce by ethylene, but they activate rather than inhibit growth. The growth of these varieties is so fast that they can keep their leaves above the water surface, avoiding the hypoxic status that would be otherwise established. Rice varieties that do not possess either the *SUB1A* or *SNORKEL* genes are still able to grow under partial submergence, thanks to the extensive aerenchyma in the rice stems and roots. However, these varieties cannot survive complete submergence.

Rice is one of the most flooding tolerant crops (Figure 1). Understanding the mechanisms that explain why rice is so well adapted to flooding has led to the identification of a group of TFs that control rice tolerance to flooding (Xu et al., 2006; Perata and Voesenek, 2007; Bailey-Serres and Voesenek, 2008). The expression of these TFs is induced by ethylene, a gaseous plant hormone that is entrapped by the water surrounding the submerged plant parts, leading to its increased tissue concentration.

Rice uses two strategies to survive submergence: “quiescence” and “escape” (Figure 1). Some indica varieties of *Oryza sativa* can tolerate flooding by applying the quiescence strategy and slowing down their metabolism and growth. This tolerance strategy is conferred by the quantitative trait locus (QTL) *SUBMERGENCE1* (*SUB1*), which contains two or three ERF genes belonging to group VII of this large family of transcription factors (Licausi et al., 2013): *SUB1A*, *SUB1B*, and *SUB1C* (Xu et al., 2006).

Ethylene accumulation in plant cells as a results of submersion induces *SUB1A* in genotypes that possess this gene (Perata and Voesenek, 2007). SUB1A induces a “quiescence” status in submerged rice plants, reducing growth and the use of carbohydrates. This ensures that the plant can preserve carbon reserves, thereby enabling re-growth of the plant when the water recedes (Perata and Voesenek, 2007).

On the other hand, an escape strategy is exploited in a different group of rice varieties, deepwater rice, which are able to grow extremely rapidly in response to submergence (Hattori et al., 2009). Ethylene is again the trigger of the response. Ethylene trapped by water surrounding the rice plant induces *SNORKEL* (*SK*) genes, which cause the stem to elongate rapidly. This results in an ‘escape’ from submergence, enabling the plant to keep its leaves above the water surface, thus allowing oxygen to be transported from the air to the submerged parts of the plant. *SNORKEL1* and *SNORKEL2* belong to the ERFVII transcription factor’s subgroup, which triggers a gibberellin-dependent internode elongation (Hattori et al., 2009). Non-deepwater rice also became deepwater rice when three quantitative trait loci from deepwater rice were introduced into them (Hattori et al., 2009).

Interestingly, rice species that do not possess either *SUB1A* or *SK* genes are intolerant to prolonged submergence. Exploitation of one of these TFs, namely, SUB1A, led to the successful development of new submergence-tolerant rice varieties (Emerick and Ronald, 2019). Introgression or overexpression of *SUB1A-1* into submergence-intolerant cultivars confers significant submergence tolerance (Fukao et al., 2006; Xu et al., 2006; Singh et al., 2009).

After the discovery of the SUB1A-dependent mechanism of submergence tolerance in rice, scientists tried to transfer this discovery to other plant species. Rice varieties possessing the *SUB1A* gene are able to survive prolonged submergence because ethylene, which is entrapped between the plant-water interface, activates the transcription of the *SUB1A* which in turns limits the response to gibberellin (Fukao and Bailey-Serres, 2008).

The ectopic expression of rice *SUB1A-1* in Arabidopsis, however, did not enhance submergence tolerance (Peña-Castro et al., 2011), indicating that the mechanism regulated by SUB1A-1 is rice-specific or, possibly, cereal-specific, a hypothesis that is awaiting experimental validation (Vasil, 2007).

The fact that SUB1A belongs to the family of Ethylene-Responsive-Factors (ERFVII) (Giuntoli and Perata, 2018) boosted research on the role of this family of TFs in other plant species, including crops and Arabidopsis. This led to the breakthrough discovery of the oxygen sensing mechanism in plants (Gibbs et al., 2011; Licausi et al., 2011).

## Oxygen sensing in plants: the N-degron pathway regulates the stability of ERFVII transcription factors

**FIGURE 2:**
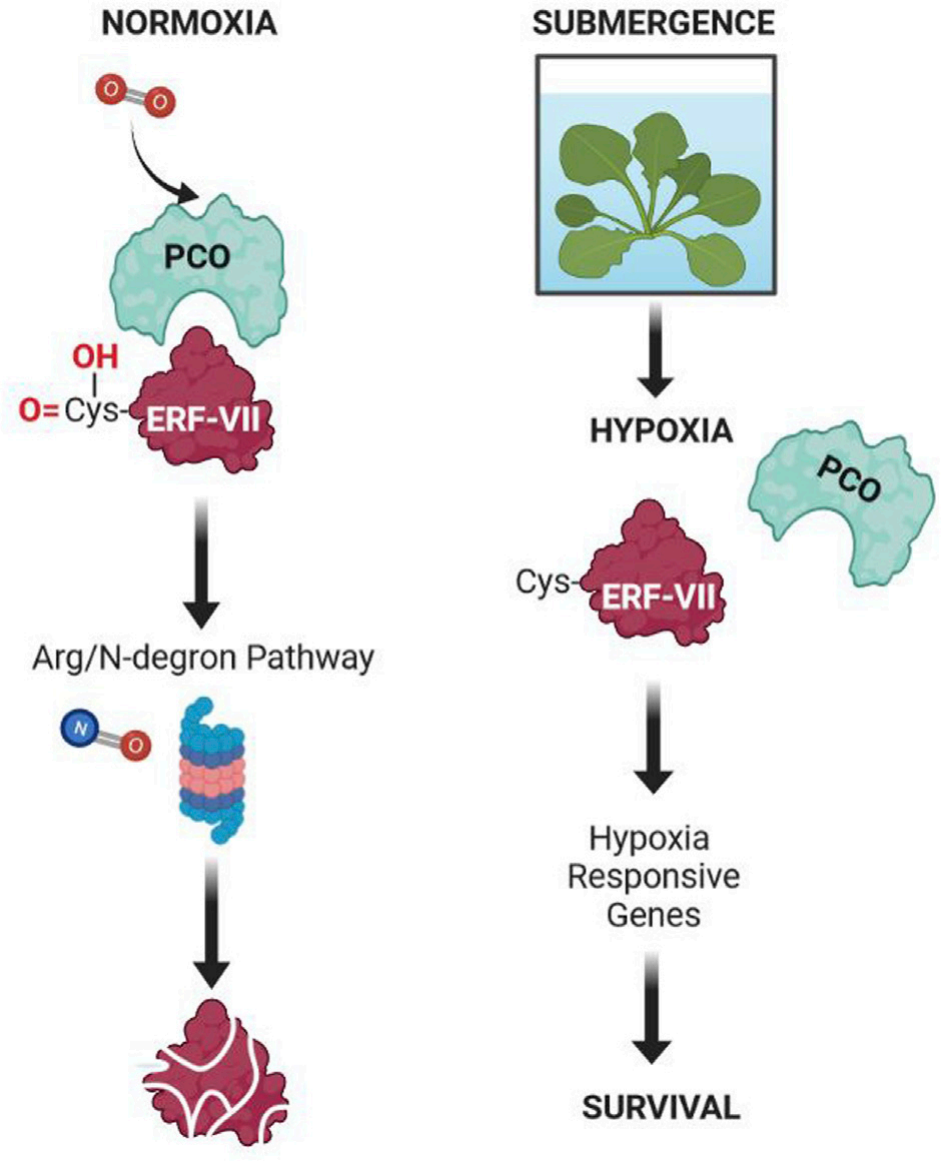
Molecular mechanism of oxygen sensing in Arabidopsis. Under normoxia, oxygen activates PCOs, which are enzymes capable of oxidizing the N-terminal cysteine residue in some of the proteins displaying a Cys residue after the removal of the N-terminal methionine. Of these proteins, ERFVII are the transcription factors directly involved in the regulation of the plant’s response to hypoxia. Although PCOs do not require nitric oxide, it is important in the overall pathway leading to the degradation of proteins displaying an oxidized Cys residue at the N-terminus. Under submergence, the level of oxygen is low (hypoxia), leading to inhibited activity of PCOs. This enables the ERFVII to be stable and to activate the transcription of HRGs, eventually leading to the acclimation to this abiotic stress. This mechanism has been demonstrated in most plant species, including crops.

The oxygen sensing mechanism has been clarified in both animals and plants, and in both cases unrelated TFs have been identified as being responsible for the perception of oxygen. In mammals the Hypoxia Inducible Factor (HIF-1) is responsible for low-oxygen sensing, while in plants the transcription factor RAP2.12 belonging to ERFVIIs is the oxygen sensor (Gibbs et al., 2011; Licausi et al., 2011), together with enzymes belonging to the family of Plant Cysteine Oxidases (PCOs) (Weits et al., 2014) (Figure 2).

The oxygen sensor in plants was discovered thanks to the model plant Arabidopsis (Gibbs et al., 2011; Licausi et al., 2011). In Arabidopsis there are five ERFVIIs members: HYPOXIA-RESPONSIVE ERFs (HRE1, HRE2), and RELATED TO APETALA2 (RAP2.2, RAP2.3, RAP2.12). They all contain the N-terminal motif [MCGGAII(A/S)D], which is characteristic of the ERFVII group (Nakano et al., 2006). Although five members of the ERFVII transcription factor exist, RAP-type ERFVII are those involved in oxygen sensing, with HRE-type ERFVII acting downstream. In normoxia RAP2.12 is associated with the plasma membrane through interaction with acyl-CoA-binding (ACBP) proteins (Licausi et al., 2011). On the other hand, during hypoxia RAP2.12 moves into the nucleus, driving the induction of hypoxia-responsive genes (HRGs) by binding an HRPE (hypoxia response promoter element) motif which is present in the promoter of hypoxia genes (Gasch et al., 2016). It is believed that the interaction with ACBP protects RAP2.12 from oxygen dependent degradation, which would occur if this protein was not docked to the plasma membrane through interaction with ACBPs (Licausi et al., 2011).

In O_2_-replete conditions, ERFVIIs are indeed easily degraded. ERFVIIs experience a series of N-terminal modifications, including N-terminal methionine excision, oxidation of the cysteine residue, and N-terminal arginylation, which boosts their degradation by the N-recognin E3 ligase PROTEOLYSIS6 (PRT6) (Gibbs et al., 2014; 2016; Weits et al., 2014; White et al., 2017). Under hypoxia, the N-terminal cysteine (after Met excision) cannot be oxidized due to the lack of oxygen and, consequently the ERFVIIs are stabilized and can drive transcriptional activation of the *HRG* genes. Oxidation of the cysteine residue does not occur spontaneously in the presence of molecular oxygen, but requires PCO enzymes, which in addition to ERFVII, can oxidize other proteins displaying a N-terminal Cys residue (Weits et al., 2014), although ERFVII and VRN2 are at present the only two confirmed PCO substrates. The stability of ERFVIIs is crucial for the plant homeostatic response to oxygen availability, regulating not only flooding survival (environmental hypoxia) but also developmental hypoxia, for example, in hypoxic niches (Gibbs et al., 2018; Weits et al., 2019). Nitric oxide is also required, though this does not seem to be via Cys oxidation and likely acts via one of the other enzymatic components of the pathway (Gibbs et al., 2014).

Oxygen sensing is also controlled by the energy sensor target of rapamycin (TOR). If hypoxia occurs in a plant with limited sugar reserves, the activity of ERFVIIs is severely dampened because the TOR controls the phosphorylation of ERFVII, which is required for optimal activity of these proteins as transcription factors (Kunkowska et al., 2023). Phosphorylation of ERVFII appears to be of great importance. In rice, mitogen-activated protein kinase3 (MPK3) is activated by submergence by SUB1A. MPK3 phosphorylates SUB1, and the tolerant allele SUB1A1 binds to the MPK3 promoter and regulates its expression in a positive regulatory loop during submergence (Singh and Sinha, 2016). One member of the calcium-dependent protein kinases (CDPKs) family in Arabidopsis, namely, CPK12, is activated during hypoxia through calcium-dependent phosphorylation of its Ser-186 residue. Phosphorylated CPK12 is then rapidly re-localized from the cytoplasm to the nucleus, where it interacts with and phosphorylates ERFVIIs to enhance their stability (Fan et al., 2023).

As previously mentioned, SUB1A plays a crucial role in submergence tolerance in rice but, despite belonging to the ERFVII class of transcription factors and showing a Cys-residue at the N-terminus, it is not a substrate for the PCO-dependent N-degron pathway (Gibbs et al., 2011). The SUB1A-1 C-terminus interacts with the SUB1A-1 N-terminus and prevents its turnover, which may explain how SUB1A-1 evades the N-degron pathway (Lin et al., 2019).

This raises the question as to how during submergence rice senses low oxygen conditions. Interestingly, SUB1A transcriptionally activates ERF66 and ERF67, which are ERFVII proteins that are substrates of the N-degron pathway and promote submergence survival (Lin et al., 2019). The overexpression of ERF66 or ERF67, actually leads to the activation of hypoxia-responsive genes (HRGs) and enhanced submergence tolerance (Lin et al., 2019). In rice, activation of the ERFVII proteins responsible for adaptation to submergence (SUB1A, SK1, SK2) is therefore hierarchically controlled by ethylene perception as a proxy for the submerged status of the plant. This is coupled with oxygen sensing that regulates the expression of ERVII (*ERF66* and *ERF67*) which controls the activation of several *HRG*s. Although plant species other than rice do not appear to have developed such a sophisticated mechanism for submergence tolerance, their response to submergence is still likely to be more complex than the response to hypoxia *per se*.

There is evidence of this also in dicots such as Arabidopsis, where ethylene is required to prime plants for the ERFVII response (Hartman et al., 2019). Ethylene entrapment triggers the expression of the nitric oxide (NO) scavenger PHYTOGLOBIN1 (PGB1), leading to rapid NO depletion, which stabilizes ERFVII proteins (Gibbs et al., 2014). This priming ensures the efficient induction of *HRGs* when hypoxia follows ethylene perception, which thus converges with oxygen sensing in order to ensure that the response is localized in the tissues that are actually experiencing hypoxia due to submergence and not developmental hypoxia (Perata, 2020).

## ERFVIIs in crop adaptation to flooding

Barley (*Hordeum vulgare* L) is very sensitive to waterlogging, resulting in a 20%–30% yield loss (de San Celedonio et al., 2014). In barley, ERFVII proteins play a crucial role in the plant’s response to waterlogging or hypoxia. Mendiondo et al. (2016) explored the potential of enhancing waterlogging tolerance in barley through the manipulation of the N-degron pathway E3 ligase *PROTEOLYSIS6* (*PRT6*) gene. They found that *PRT6* plays a crucial role in regulating the plant’s response to waterlogging stress. The barley ERFVII *BERF1*, most closely related in sequence to Arabidopsis RAP2.12, is a substrate of the N-degron pathway *in vitro.* By reducing the expression of *PRT6* in barley plants, the authors were able to enhance the plant’s waterlogging tolerance, presumably because the lower level of *PRT6* expression leads to a higher stability of ERFVII proteins and thus increased expression of hypoxia-associated genes. The study suggests that manipulating the expression of *PRT6* in crops could be a promising strategy for improving waterlogging tolerance.

Maize is very sensitive to waterlogging stress. Waterlogging severely affects approximately 18% of the land in south and south-east Asia, and annually results in production losses of 25%–30% (Zaidi et al., 2015). Nineteen ERFVIIs of maize (ZmERFVIIs) have been identified and characterized (Yu et al., 2019). Most of these ZmERFVIIs share the conserved N-terminal motif, suggesting that similar functions to ERFVIIs in rice and Arabidopsis may also exist. A candidate gene association analysis of the ZmERFVII gene family showed that a waterlogging-responsive gene, ZmEREB180, was tightly associated with waterlogging tolerance. Transgenic Arabidopsis overexpressing *ZmEREB180* showed significantly enhanced submergence tolerance in comparison with the wild type. Anaerobic metabolism genes (*AtADH1*, *AtPDC1*, *AtSUS1*, and *AtSUS4*) were significantly upregulated in the *ZmEREB180* transgenic plants compared with WT under submerged conditions (Yu et al., 2019). Similar results were obtained by overexpressing *ZmEREB180* in transgenic maize seedlings (Yu et al., 2019). These results indicated that overexpression of *ZmEREB180* confers tolerance to waterlogged conditions in maize plants, suggesting that ERFVIIs are involved in defining the responses of maize plants to limited oxygen availability (Yu et al., 2019).

In wheat, another crop which is very sensitive to waterlogging, 25 ERFVII genes have been identified (Wei et al., 2019). To enhance waterlogging tolerance in wheat, transgenic plants have been developed that express a stabilized form of an ERFVII gene under the control of a constitutive promoter. The stabilized ERF protein is more stable than the wild-type protein, leading to higher levels of ERF activity in the transgenic plants. Transgenic wheat plants with constitutive expression of the stabilized ERFVII gene TaERFVII.1 have been shown to promote better tolerance to waterlogging than non-transgenic plants (Wei et al., 2019). The transgenic plants show improved root growth, increased expression of genes involved in anaerobic respiration, and reduced accumulation of reactive oxygen species (ROS) under waterlogged conditions (Wei et al., 2019). Importantly, these plants do not show any significant reduction in grain yield under normal growth conditions (Wei et al., 2019).

Soybean is extremely sensitive to waterlogging (Linkemer et al., 1998). In soybean, nine genes encoding for ERFVII proteins (ERFVII 1–9) were identified. Of these, four were highly induced by submergence and downregulated during the recovery phase. In addition, ERFVII1, ERFVII5, and ERFVII8 showed a strong induction in response to ACC, the precursor of ethylene, suggesting that these three genes are possible candidates for the response of soybean to submergence tolerance (Tamang et al., 2014). In contrast, ERFVII2, ERFVII3, ERFVII7, and ERFVII9 were not upregulated during flooding or modulated during reoxygenation. One phylogenetic analysis highlighted that the soybean ERFVII proteins that respond to submergence (ERFVII1, ERFVII5, and ERFVII8) are related to HRE2 an Arabidopsis ERFVII gene that is strongly upregulated under hypoxia. On the other hand, ERFVII3, ERFVII7, and ERFVII9 are similar to Arabidopsis RAP2.2 and RAP2.12, whereas ERFVII4 and ERFVII6 are homologous of RAP2.3, which are not regulated by hypoxia at the transcriptional level but help Arabidopsis plants to adapt to low oxygen. This is due to their hypoxia-dependent stabilization, as described above, which suggests that these ERFVIIs in soybean could play a role in the submergence response and tolerance via the N-degron pathway (Tamang et al., 2014).

## Transcriptional regulation of the hypoxic response: beyond abiotic stress tolerance

In addition to hypoxia caused by adverse environmental conditions such as flooding events in the plant kingdom, physiological hypoxia can also occur in tissues and organs under normal oxygen levels. The anatomy of some organs such as seeds, fruits and roots do not facilitate the diffusion of oxygen (Van Dongen et al., 2003; Borisjuk and Rolletschek, 2009; Ho et al., 2010). The oxygen concentration was found to be very low in the inner part of bulky organs such as potato tubers when the oxygen concentration reaches 5% therefore making it hypoxic (Geigenberger et al., 2000). Dormant or quiescent buds of woody perennials are also often very compact, which may lead to internal hypoxia, as shown in the case of grapevine (*Vitis vinifera* L.) (Meitha et al., 2018). Interestingly, the three ERFVIIs in grapevine were substrates for oxygen-dependent proteolysis *in vitro*, because of the N-terminal cysteine (Meitha et al., 2018).

Hypoxic niches can affect plant development and can exist in otherwise aerobic plants (Kerpen et al., 2019; Weits et al., 2019; Loreti and Perata, 2020; Valeri et al., 2020). The shoot apical meristem is hypoxic and the existing oxygen gradients likely act as positional cues that guide the plant’s development (Weits et al., 2020). Other plant tissues that are physiologically hypoxic include the lateral root primordia (Eysholdt-Derzsó and Sauter, 2017; Shukla et al., 2019) and the anthers (Kelliher and Walbot, 2014). In these tissues, hypoxia does not merely arise due to the tissue thickness that limits oxygen diffusion as in bulky fruits or tubers (van Dongen and Licausi, 2015). Instead, it appears to be generated deliberately by a still unknown process, restricting the presence of specific, oxygen sensitive proteins to only the hypoxic niche.

The contribution of ERFVII proteins in hypoxic niches is under investigation. In Arabidopsis during *Botrytis* infection, local hypoxia was established allowing the stabilization of ERFVII proteins (Valeri et al., 2020). The results suggest that hypoxia was generated at the site of the infection which may affect the stability of N-degron regulated proteins.

Regarding the hypoxic niches that are generated during development, ZPR2 (Weits et al., 2019) and VRN2 (Gibbs et al., 2018; Labandera et al., 2021) are two proteins whose stability depends upon hypoxia via the Cys-branch of the N-degron pathway. They are involved in new leaf production and in the transition to flowering, respectively. Notably, both these proteins are substrates of the PCO-dependent N-degron pathway, and since they are oxygen labile, they are present in an active form only in a hypoxic environment.

## Concluding remarks

The outstanding work carried out in rice, in which the discovery of SUB1A as the gene responsible for this trait was rapidly translated from the lab to the field, demonstrating that a clever combination of genetics with physiology can produce breakthroughs in agriculture. Since the discovery of SUB1A (Fukao et al., 2006; Xu et al., 2006) and other ERFVII as transcriptional regulators of rice adaptation to submergence (Hattori et al., 2009), research on this group of ERFs has flourished (Licausi et al., 2013; Giuntoli and Perata, 2018). The discovery of the oxygen sensing of plants was a breakthrough in plant hypoxia research (Gibbs et al., 2011; Licausi et al., 2011). The discovery that ERFVII are targets of the N-degron pathway, and as PCOs are able to define the fate of proteins displaying a N-terminal MC motif (MC proteins) (Weits et al., 2014), led to the search for other MC proteins that could be substrates for PCOs and, therefore, represent oxygen-sensitive proteins that are stable only under low oxygen conditions. There are more than 200 putative MC-proteins in the Arabidopsis genome and, although not all of them are actual substrates for the PCO-dependent branch of the N-degron pathway, some, such as ZPR2 and VRN2 are key to plant development (Gibbs et al., 2018; Weits et al., 2019). In addition, hypoxic niches seem to be important determinants of plant growth and development (Weits et al., 2020).

The existence of hypoxic niches also during plant-microbe interactions is another promising research area, which will help in highlighting the importance of oxygen-labile transcription factors that influence plant life (Kerpen et al., 2019a; Valeri et al., 2020; Xu et al., 2023).

After the identification of the SUB1A gene, despite the success of the new rice varieties that were highly tolerant to flooding, there have been few positive results in translating the discovery of oxygen sensing into new crop varieties. One likely explanation for this is that, unlike SUB1A, the regulation of oxygen sensing following a flooding event is shared by hypoxic niches that control developmental processes. One outstanding challenge is to uncouple the responses to flooding conditions from those linking hypoxia to plant growth and development in order to make biotechnological approaches toward flooding-tolerant plants feasible.
